# Practical Notes and Formulæ

**Published:** 1887-09

**Authors:** 


### PRACTICAL NOTES AND FORMULÆ.

**For Cholera, Diarrhœa, etc.**—

℞. Spirits camphor........................
Oil of turpentine........................
Tincture opium...................... ....aaʒ vi,
Oil of peppermint...........................gtts. c.

M. Dose, 1 teaspoonful in 2 of brandy, repeated at intervals of one hour if necessary.

**For Catarrhal Conjuntivitis.**—

℞. Sodæ biboratis............................grs. xx,
Aq. camphoræ.............................f ℥ jss,
Mucil. cydonii sem..........................
Aq. laurocerasi.........................aa f ℥ ss

M. Sig.—Apply to lids and drop in eyes three times daily.

**Syrup Doveri** made after the following formula will keep indefinitely:

℞. Sulphate of potash....................℥ j. grs. xxxii,
Hot water.............................O j,
Sugar.........................℥ xij,
Tr. opium (U. S. P. 1870)..............℥ jss,
Fl. ext. ipecac.........................♏ lxiv.

Dissolve the sugar and potash in the hot water and evaporate to 15 fluid ounces, strain, cool, and add the opium and ipecac.—*Indiana Medical Journal.*

**For Clergyman's Sore Throat.**—

R. Tinct. guaiaci ammon.......................

Liq. potassæ...............................aa f ʒ iij,

Tinct. opii............................... f ʒ ij,

Aquæ cinnamoni, ad....................... f ℥ viij.

M. Sig.—Used every hour, as a gargle.—*Ib.*

**Nerve Tonic in Opium Cases.**—

℞. Tinct. nucis vomicæ.........................gtts. xij,
Acidi phosphorici dil........................gtts. xx,
Syr. pruni Virg.............................f ℥ ss.

M. Sig.—To be taken twice daily.—*Ib.*

**Singultus.**—A positive but empirical remedy—tr. turmeric *ad lib.*—*Western Medical Reporter.*

**Puerperal Convulsions.**—Dr. Baird, of Texas (in *Medical Brief*), highly recommends the following: Place a cork or some other material between the teeth, to prevent patient from wounding tongue with the teeth during convulsions. Mix up mustard with vinegar, and apply to wrists and ankles and along the spine, and administer by mouth the following:

℞. Tinct. lobeliæ.................................fl ℥ j,

Tinct. ictodes fœtidi................................fl ℥ j,

Tinct. capsici................................fl ℥ ss.

M. Sig.—Give a teaspoonful in simple syrup or sweet milk every fifteen minutes till the paroxysms are shortened and the intervals are longer between the paroxysms, then repeat every two or three hours as symptoms indicate.

Follow this when convulsions are stopped, with quinine, bromide potassium and fl. ext. valerian for a day or two.

I have been universally successful with the above treatment. It is also good in infantile convulsions.

**Diphtheria.**—Dr. Moore (*Medical Brief*) uses with great success the following: Mop the throat with nitrate of silver and use the following gargle, if the patient is old enough to gargle his throat, every half hour; if not old enough, let him swallow from ½ to 1 teaspoonful:

℞. Potass. chlor................................ʒ j,

Powd. hydrastis can................................ʒ j,

Tinct. myrrh................................ʒ ij,

Powd. capsici................................grs. iv,

Aquæ................................℥ vi.

M.

If the bowels are torpid, I give small doses of calomel and bicarb. soda, every three hours, till they move freely. I apply cotton-seed poultices; bruise the seed well, and boil half an hour; thicken with a little cornmeal, and put in a small sack and apply to the throat, cool; grease the side with lard that goes next to the skin, and renew every five hours. When the throat is about well, I give quinine and elix. vitriol every three hours, for a few days, to prevent a backset.

**Diarrhœa.**—Pichini successfully treated eight cases, with signs of fermentation, with the following:

℞. Iodoform................................grs. ix,

Ether................................℥ 3⅛,

Vegetable charcoal, finely powdered........℥ 3⅛,

Glycerine, ad................................℥ xii.

Dissolve the iodoform in the ether; thoroughly mix in the powdered charcoal; add glycerine after ether has evaporated. Teaspoonful or tablespoonful every twenty-four hours in a glass of water.—*Journal de Medecine.*

**Scabies.**—(Kaposi.)—Of the numerous remedies one of the best is the modified Wilkinson's ointment:

℞. Flor. sulphur ........................
Rusci ........................ aa 20 parts,
Sapon. virid ........................ 40 parts,
Pulv. creta alb ........................ 5 parts,
Adipis ........................ 40 parts.

After many experiments with others, we have always returned to this remedy.—*Wiener Medical Zeitung.*

**Constipation.**—The Memphis *Medical Monthly* gives the following formula for artificial Hunyadi Janos water for constipation:

℞. Magnesii sulphatis ........................
Sodii sulphatis ........................ aa℥ ss,
Potassii sulphatis ........................ grs. ij,
Sodii bicarbonatis ........................ grs. viij,
Sodii chloridi ........................ grs. xx,
Aquæ, ad ........................ ℥ viij.
M. Sig.—Wineglass before breakfast.—*Ib.*

**A Good Cholagogue Pill.**—

℞. Podophyllin ........................ gr. v,

Leptandrin ........................

Caulophyllin ........................ aa grs. viij.

Ipecacuanhæ ........................ grs. ij.

M. ft. pil. no. viij. Dose, 1 to 2 pills.—*Medical Record.*

**For Summer Diarrhœa.**—

℞. Tinct. opii deodorat ........................ gtt. vj,
Tinct. catechu ........................ f ʒ jss,
Syr. rubi villosi ........................
Syr. rhei aromat ........................ aa f ʒ iijss,
Aq. camphoræ ........................ f ℥ j.

M. S.—A teaspoonful every hour or two, for a child under one year.—*Ib.*

**For Vomiting of Cholera Infantum.**—

℞. Bismuth subnit ........................ grs. x,
Mucil. acaciæ ........................ f ʒ ss,
Acid. carbolici ........................ gr. 1-12,
Tinct. opii deodorat ........................ gtt. j,
Mist. cretæ ........................ f ʒ jss.

M. S.—To be given every two hours, for a child one or two years old.—*Ib.*

**Neuralgia.**—Dr. J. P. Hillegass, of Pennsburg, Pa., writes approvingly of the mixture of chloral and camphor, equal parts, painted over the affected nerves. We have used this remedy and found it a good one.

